# Concordance of Extent of Resection Between Intraoperative Ultrasound and Postoperative MRI in Brain and Spine Tumor Resection

**DOI:** 10.7759/cureus.74101

**Published:** 2024-11-20

**Authors:** Enrique Caro-Osorio, Luis Alejandro Perez-Ruano, Jose Antonio Figueroa Sanchez

**Affiliations:** 1 Neurosurgery, Tecnologico de Monterrey, Monterrey, MEX

**Keywords:** brain tumor, intraoperative ultrasound, magnetic resonance image, neurosurgery, oncology, spine tumor, tumor resection

## Abstract

Objective

Evaluate the utility of intraoperative ultrasound (IOUS) in brain and spinal tumor resections and the concordance of the extent of resection between IOUS and postoperative magnetic resonance imaging (MRI).

Methods

A retrospective analysis of prospectively collected data was performed. Thirty-nine lesions (36 patients) in the brain and spine were operated on for resection using IOUS between May 2020 and December 2022. All patients who underwent brain or spinal tumor resection were included, and who underwent IOUS during tumor resection and postoperative MRI was done within 48 hours of surgery. The Cohen's kappa coefficient was performed to the concordance of resection by IOUS and postoperative MRI.

Results

Forty-one patients underwent surgery, of which 36 met the inclusion criteria and five were excluded due to incomplete clinical records. Of the 36 patients included, two presented lesions in different locations, one with frontal and parietal metastases and the other with extradural and intradural spinal lesions, and one patient had a recurrence of glioblastoma, for which 39 procedures were included. Of the 36 patients, 36% were women, and the average age was 51.4 years with an age range of one year and two months to 94 years. The concordance of the degree of resection by ultrasound and the degree of resection by postoperative magnetic resonance is high.

Conclusions

The higher degree of concordance between IOUS and postoperative MRI would suggest that IOUS is a reliable tool for assessing the extent of tumor resection during surgery and provides real-time information to make decisions to adjust surgery plans based on the benefit/risk ratio.

## Introduction

The neuro-oncologists' determination to achieve a higher degree of resection with a low risk of neurological damage has led to a better understanding of tumor biology, better preoperative studies, better-developed skills of spatial memory, mental recognition, orientation in a 3D topography [1], and development of intraoperative real-time imaging tools like intraoperative magnetic resonance imaging (iMRI), CT and ultrasound (intraoperative ultrasound (IOUS)). During tumor resection, all these aspects are important to accurately locate the normal brain structures and delimited lesions with a low risk of error. iMRI is the gold standard to describe brain tumors, and it permits achieving gross total resection (GTR) but is very expensive, logistically difficult to do during surgery, needs a neuroradiologist, consumes approximately 50-90 min of surgery time, and is not available in all centers [2-6].

IOUS is one of the most useful tools for increasing the degree of resection, thereby improving progression-free survival and overall survival [7,8]. Also, it was a tool rarely used in neurosurgery, which may be due to the lack of expertise and the lack of standardized protocols, but in the last years, it has been popularized and developed by dedicated brain US machines with neuronavigation interfaces [9-11]. Several authors observed A high concordance between iOUS and post-operative MRI in assessing the degree of resection [12]. Ivanov et al. described IOUS's advantages and disadvantages compared to iMRI. We considered the most important are the real-time information, easily repeatable, rapid intraoperative availability, no irradiation, and low cost [13]. In this study, we evaluated the agreement between these two imaging modalities to determine how accurate IOUS is at predicting the extent of brain tumor resection compared to postoperative MRI.

## Materials and methods

Study design

A retrospective analysis of prospectively collected data was performed. The study was approved by the internal institutional review board (Institutional Review Board of Tecnologico de Monterrey, Monterrey, Mexico). Thirty-nine cases of surgical resection of brain and spinal tumors were performed between May 2020 and December 2022. IOUS was used and an MRI was done within 48 hours postoperatively in all cases. The ultrasound equipment used was Philips Ultrasound CX50 Compact Xtreme (Philips, Eindhoven, Netherlands) in the first 15 subjects. Then, we had access to bk5000 (EC1) Medical Ultrasound (BK Medical, Burlington, MA) for the rest of the cases. A standard surgery was planned, reviewing the MRI to decide the route of approach. IOUS was used according to the protocol described by Prada et al. [14]. A scan is made before opening the dura, and immediately after that. At least two orthogonal planes were obtained, and a slipped exploration of the lesion and anatomy is registered on each step, and the same maneuvers are done during and, at the end of the procedure, by the same neurosurgeon. When Philips Ultrasound CX50 CompactXtreme was used, the probe was draped in a sterile cover filled with sterile jelly, and all air bubbles were eliminated. When bk5000 Ultrasound was used, there was no need to drape the probe because it could be sterilized. Inclusion criteria were patients undergoing surgery at Hospital Zambrano-Hellion with a diagnosis of neoplastic brain lesions, non-neoplastic brain lesions such as dermoid cysts, epidermoid cysts, and angiomas, spinal tumors, and patients with a complete clinical record. Exclusion criteria were patients diagnosed with traumatic, infectious, infestations, ischemic, demyelinating, and vascular brain lesions (except cavernous malformations) and incomplete medical records. The Cohen's kappa coefficient was performed to the concordance of resection by IOUS and postoperative MRI performed within 48 hours of surgery. The extent of resection was described according to the standard agreement of 100% for gross total, >90% near-total, 51%-90% subtotal and 10%-50% for partial resection. For our study, we consider subtotal and near-total as subtotal as a whole.

Data analysis

Descriptive statistics were used including mean, proportions, and distribution. To evaluate the utility of IOUS, Cohen's kappa coefficient was performed to establish the expected concordance and the actual concordance for total, subtotal, and partial resection by IOUS and postoperative magnetic resonance imaging. The value of P < 0.05 indicates that the degree of agreement is sufficient to reject the hypothesis that the agreement between the two methods is random.

## Results

Forty-one patients underwent surgery, of which 36 met the inclusion criteria and five were excluded due to incomplete clinical records. Of the 36 patients included, two presented lesions in different locations, one with frontal and parietal metastases, the other with extradural and intradural spinal lesions, and one patient had a recurrence of glioblastoma, for which 39 procedures were included. Of the 36 patients, 13 (36%) were women, the average age was 51.4 years with an age range of one year and two months to 94 years (Table 1).

**Table 1:** Patient data BC Metastasis: breast cancer metastasis, PC metastasis: prostate cancer metastasis, SMCS: supplementary motor cortex syndrome, Paresth: Paresthesia, LCLC: large cell lung cancer, EH: epidural hematoma, Pnemph: Pneumocephalus, IE: intracranial hematoma, VFD: visual field defect, CSFF: cerebrospinal fluid fistula

Lesion	Sex	Age	Tumor	Symptom	Site	Tissue type	Echogenicity	Infiltration area	Mair	Extent of resection by IOUS	Extent of resection by MRI	Complications
1	F	72	Astrocytoma Grade 3	Hypoesthesia	Parietal	Cystic	Hypoechoic	Sensitive	III	Subtotal	Subtotal	None
2	M	61	LCLC metastasis	Seizure	Parietal	Solid	Hyperechoic	No	III	Total	Total	None
3	M	61	LCLC metastasis	Seizure	Frontal	Cystic	Hypoechoic	No	III	Total	Total	None
4	M	51	Diffuse astrocytoma	Syncope	Parietal	Solid	Hyperechoic	Sensitive	III	Total	Total	None
5	F	63	Meningioma	Paresthesia	Frontal	Solid/cystic	Hypoechoic/Hyperechoic	Si	III	Partial	Partial	None
6	M	85	Glioblastoma	Afasia	Frontal	Solid/cystic	Hypoechoic/hyperechoic	Premotor	III	Total	Total	SMCS
7	F	54	Glioblastoma	Paresthesia	Parietal	Solid/cystic	Hypoechoic/hyperechoic	Motor	III	Total	Total	Paresth
8	F	36	Cavernous malformation	Seizure	Temporal	Solid	Hyperechoic	No	III	Total	Total	None
9	M	71	Glioblastoma	Headache	Frontal	Solid/cystic	Hyperechoic	Premotor	III	Partial	Partial	None
10	M	62	Meningioma	Paresthesia	Frontal	Solid	Isoechoic	No	III	Partial	Partial	None
11	F	39	BC metastasis	Sensory ataxia	Parietal	Solid/cystic	Hypoechoic/hyperechoic	Sensitive	III	Total	Subtotal	None
12	F	64	Meningioma	Paresthesia	C1-C7	Solid	Hyperechoic	No	III	Partial	Partial	C5 Paresth
13	M	65	Glioblastoma	Disorientation	Temporal	Solid/cystic	Hypoechoic/hyperechoic	No	II	Total	Total	None
14	M	39	Glioblastoma	Seizure	Temporal	Solid/cystic	Hypoechoic/hyperechoic	No	III	Partial	Partial	None
15	M	1	Mature teratoma	Paraplegia	T4-T6	Solid	Hyperechoic	No	III	Total	Total	None
16	M	1	Mature teratoma	Paraplegia	T4-T6	Solid/cystic	Isoechoic	No	III	Total	Total	None
17	M	94	Glioblastoma	Hemiplegia	Frontal	Solid/cystic	Hypoechoic/hyperechoic	No	II	Subtotal	Subtotal	EH
18	F	58	Meningioma	Headache	Frontal	Solid	Hyperechoic	No	III	Subtotal	Subtotal	None
19	M	39	Glioblastoma	Progression	Temporal	Solid/cystic	Hypoechoic/hyperechoic	No	III	Partial	Partial	None
20	M	72	LCLC metastasis	Seizure	Frontal	Solid/cystic	Hypoechoic/hyperechoic	No	III	Partial	Partial	Pnemph
21	M	13	Cavernous malformation	Seizure	Temporal	Solid	Hyperechoic	No	III	Total	Total	None
22	F	30	Cavernous malformation	Seizure	Temporal	Solid	Hyperechoic	No	III	Total	Total	None
23	M	79	Meningioma	Hemiparesis	Frontal	Solid	Isoechoic	No	III	Total	Total	None
24	F	49	Glioblastoma	Headache	Temporal	Solid/cystic	Hypoechoic/hyperechoic	No	III	Subtotal	Subtotal	None
25	M	79	Glioblastoma	Amnesia	Frontal	Solid/cystic	Hypoechoic/hyperechoic	No	III	Partial	Partial	IH
26	M	39	Glioblastoma	Progression	Temporal	Solid/cystic	Hypoechoic/hyperechoic	No	III	Subtotal	Subtotal	VFD
27	F	30	Low grade glioma	Seizure	Frontal	Solid	Hyperechoic	Si	III	Partial	Partial	None
28	M	21	Medulloblastoma	Neck pain	Cervical	Solid	Isoechoic	No	III	Partial	Partial	None
29	F	45	BC metastasis	Seizure	Parietal	Solid/cystic	Hypoechoic/hyperechoic	Sensitive	III	Partial	Partial	Edema
30	M	67	Glioneuronal tumor	Headache	Parietal	Solid	Hyperechoic	Sensitive	III	Total	Total	Seizure
31	M	39	Glioblastoma	Disorientation	Temporal	Solid/cystic	Hypoechoic/hyperechoic	No	III	Partial	Partial	Paresth
32	F	60	LCLC metastasis	Dysphasia	Parietal	Solid/cystic	Hypoechoic/hyperechoic	Sensitive	III	Total	Total	None
33	M	49	Glioblastoma	Dysmetria	Parietal	Solid/cystic	Hypoechoic/hyperechoic	Sensitive	III	Partial	Partial	None
34	M	61	Glioblastoma	Seizure	Parietal	Solid/cystic	Hypoechoic/hyperechoic	Sensitive	III	Partial	Partial	None
35	M	15	Astrocytoma Grade 3	Seizure	Parietal	Solid/cystic	Hypoechoic/hyperechoic	Sensitive	III	Subtotal	Subtotal	None
36	M	47	Meningioma	Blindness	Frontal	Solid	Hyperechoic	No	III	Total	Total	CSFF
37	M	75	PC metastasis	Paraplegia	T4	Solid	Hyperechoic	No	III	Partial	Partial	None
38	F	41	LCLC metastasis	Dysphasia	Parietal	Solid/cystic	Hypoechoic/hyperechoic	No	III	Total	Total	None
39	F	81	Meningioma	Quadrantopia	Occipital	Solid	Hyperechoic	Visual	III	Partial	Partial	None

In 13 cases (33.3%), glioblastoma was the most common brain lesion. As indicated in Table 2, other common brain lesions included eight metastases (20.5%), seven meningiomas (17.9%), three cavernous malformations (7.7%), two astrocytomas grade 3 (5.1%), two diffuse astrocytomas (5.1%), two spine teratomas (5.1%), one glioneuronal tumor (2.6%), and one medulloblastoma (2.6%).

**Table 2:** Histologic diagnosis of the lesions The data have been represented as number (N) and percentage (%).

Diagnosis	N= 39, 100%
Glioblastoma	13 (33.3)
Metastasis	8 (20.5)
Meningioma	7 (17.9)
Cavernous malformation	3 (7.6)
Astrocytoma Grade 3	2 (5.1)
Diffuse astrocytoma	2 (5.1)
Spine teratoma	2 (5.1)
Glioneuronal tumor	1 (2.5)
Medulloblastoma	1 (2.5)
Total	39 (100)

Table 3 shows that parietal lesions occurred in 13 instances (33.3%), followed by frontal lesions in 12 cases (30.7%), temporal lesions in nine cases (23.1%), spinal lesions in four cases (10.2%), and one occipital lesion (2.5%).

**Table 3:** Localization by type of lesion The data have been represented as number (N) and percentage (%).

Localization	High-grade glioma	Metastasis	Meningioma	Low-grade glioma	Cavernous malformation	Teratoma	Glioneuronal tumor	Medulloblastoma	Total (N=39, 100%)
Frontal	4 (10.2%)	2 (5.1%)	5 (12.8%)	1 (2.5%)	0 (0%)	0 (0%)	0 (0%)	0 (0%)	12 (30.7)
Parietal	5 (12.8%)	6 (15.3%)	0 (0%)	1 (2.5%)	0 (0%)	0 (0%)	1 (2.5%)	0 (0%)	13 (33.3)
Temporal	6 (15.3%)	0 (0%)	0 (0%)	0 (0%)	3 (7.6%)	0 (0%)	0 (0%)	0 (0%)	9 (23.0)
Spine	0 (0%)	0 (0%)	1 (2.5%)	0 (0%)	0 (0%)	2 (5.1%)	0 (0%)	1 (2.5%)	4 (10.2)
Occipital	0 (0%)	0 (0%)	1 (2.5%)	0 (0%)	0 (0%)	0 (0%)	0 (0%)	0 (0%)	1 (2.5)
Total	15 (38.4%)	8 (20.5%)	7 (17.9%)	2 (5.1%)	3 (7.6%)	2 (5.1%)	1 (2.5%)	1 (2.5%)	39 (100)

The Mair classification was used for localization of lesion and margin delineation [15]. Thirty-seven of the lesions correspond to grade 3 (94.8%) and two lesions to grade 2 (5.2%). In this series of cases, all the lesions were visualized by ultrasound and the edges were identified as either clearly or partially (Table 4).

**Table 4:** Mair classification The data have been represented as number (N) and percentage (%).

Grade	(N=39, 100%)
0	0 (0)
1	0 (0)
2	2 (5.2)
3	37 (94.8)

The extent of resection of each lesion was established by IOUS at the end of the procedure (Table 5), being the glioblastoma the most frequently resected partially. Because of its malignant and infiltrating nature, the surgeon can decide to stop the resection, even if the IOUS imaging shows residual tumor. On the other hand, cavernous malformation was resected totally given that it is a benign and very well delineated lesion. This result reflects that sometimes it is possible to the surgeon to see the residual tumor (partial or subtotal) and decide to leave it in. Other times, it is difficult to find the borders and important adjacent structures.

**Table 5:** Extent of resection by IOUS The data have been represented as Number (N) and Percentage (%). IOUS - intraoperative ultrasound

Diagnosis	Extent of resection by IOUS			
Histopahology	Total	Subtotal	Partial	Total
Glioblastoma	3 (23%)	3 (23%)	7 (53.8%)	13 (100%)
Metastasis	5 (62.5%)	0 (0%)	3 (37.5%)	8 (100%)
Meningioma	2 (28.5%)	1 (14.2%)	4 (57.1%)	7 (100%)
Cavernous malformation	3 (100%)	0 (0%)	0 (0%)	3 (100%)
Astrocytoma grade 3	0 (0%)	2 (100%)	0 (0%)	2 (100%)
Diffuse astrocytoma	1 (50%)	0 (0%)	1 (50%)	2 (100%)
Teratoma	2 (100%)	0 (0%)	0 (0%)	2 (100%)
Glioneuronal tumor	1 (100%)	0 (0%)	0 (0%)	1 (100%)
Medulloblastoma	0 (0%)	0 (0%)	1 (100%)	1 (100%)
Total	17 (43.5%)	6 (15.3%)	16 (41%)	39 (100%)

By MRI within 48 hours after surgery, the extent of resection was total in 16 patients, subtotal in seven patients and partial in 16 patients, representing 41%, 17.9%, and 41%, respectively. The results of extent of resection by histology are summarized in Table 6.

**Table 6:** Extent of resection by MRI The data have been represented as number (N) and percentage (%).

Diagnosis	Extent of resection by MRI				
Histopathology	Total	Subtotal	Partial		Total
Glioblastoma	3 (23%)	3 (23%)		7 (53.8%)	13 (100%)
Metastasis	4 (50%)	1 (12.5%)		3 (37.5%)	8 (100%)
Meningioma	2 (28.5%)	1 (14.2%)		4 (57.1%)	7 (100%)
Cavernous malformation	3 (100%)	0 (0%)		0 (0%)	3 (100%)
Astrocytoma Grade 3	0 (0%)	2 (100%)		0 (0%)	2 (100%)
Diffuse astrocytoma	1 (50%)	0 (0%)		1 (50%)	2 (100%)
Teratoma	2 (100%)	0 (0%)		0 (0%)	2 (100%)
Glioneuronal tumor	1 (100%)	0 (0%)		0 (0%)	1 (100%)
Medulloblastoma	0 (0%)	0 (0%)		1 (100%)	1 (100%)
Total	16 (41%)	7 (17.9%)		16 (41%)	39 (100%)

Among the observations of the degree of total resection by IOUS and postoperative contrasted enhanced magnetic resonance imaging, the expected agreement is 24.49% and the actual agreement was 85.7% with a Kappa coefficient of 0.8108 (p = 0.0001). The value of P < 0.05 indicates that the degree of agreement is sufficient to reject the hypothesis that the agreement between the two methods is random (Table 7).

**Table 7:** Intraoperative ultrasound and magnetic resonance imaging agreement for total resection The data have been represented as K = Cohen's Kappa, SE = Standard error, Z = standard score and p-value (p<0.05).

Agreement	Expected agreement	K	SE	Z	P
85.71%	24.49%	0.8108	±0.2013	3.85	0.0001

Among the observations of the degree of subtotal resection by IOUS and postoperative contrasted enhanced magnetic resonance imaging, the expected agreement is 53.06% and the actual agreement was 85.71% with a Kappa coefficient of 0.6957 (p = 0.0267). The value of P < 0.05 indicates that the degree of agreement is sufficient to reject the hypothesis that the agreement between the two methods is random (Table 8).

**Table 8:** Intraoperative ultrasound and magnetic resonance imaging agreement for subtotal resection The data have been represented as K = Cohen's Kappa, SE = Standard error, Z = standard score and p-value (p<0.05).

Agreement	Expected agreement	K	SE	Z	P
85.71	53.06	0.6957	±0.36	1.93	0.0267

Between the observations of the degree of partial resection by IOUS and postoperative contrasted enhanced magnetic resonance imaging, there is an expected agreement of 42.86% and the actual agreement was 100% with a Kappa coefficient of 1 (p = 0.0002). The value of P < 0.05 indicates that the degree of agreement is sufficient to reject the hypothesis that the agreement between the two methods is random (Table 9).

**Table 9:** Intraoperative ultrasound and magnetic resonance imaging agreement for partial resection The data have been represented as K = Cohen's Kappa, SE = Standard error, Z = standard score and p-value (p<0.05).

Agreement	Expected agreement	K	SE	Z	P
100%	42.86%	1	±0.2857	3.5	0.0002

## Discussion

Neuro-oncologists strive to achieve the best possible outcome for patients with brain tumors, including improved quality of life, longer survival, reduced risk of neurological damage, and reduced risk of recurrence. In neurosurgical procedures, maximal safe resection is the most important objective. To achieve that, neurosurgeons implement intraoperative tools, the most valuable is iMRI, which provides high-resolution images of the brain and surrounding tissue and is used to guide the surgeon during the procedure but is more expensive and sometimes it requires the patient to be moved from the operating room to the MR suite, which can be time-consuming, increase the risk of infection and is not available in all centers.

**Figure 1:**
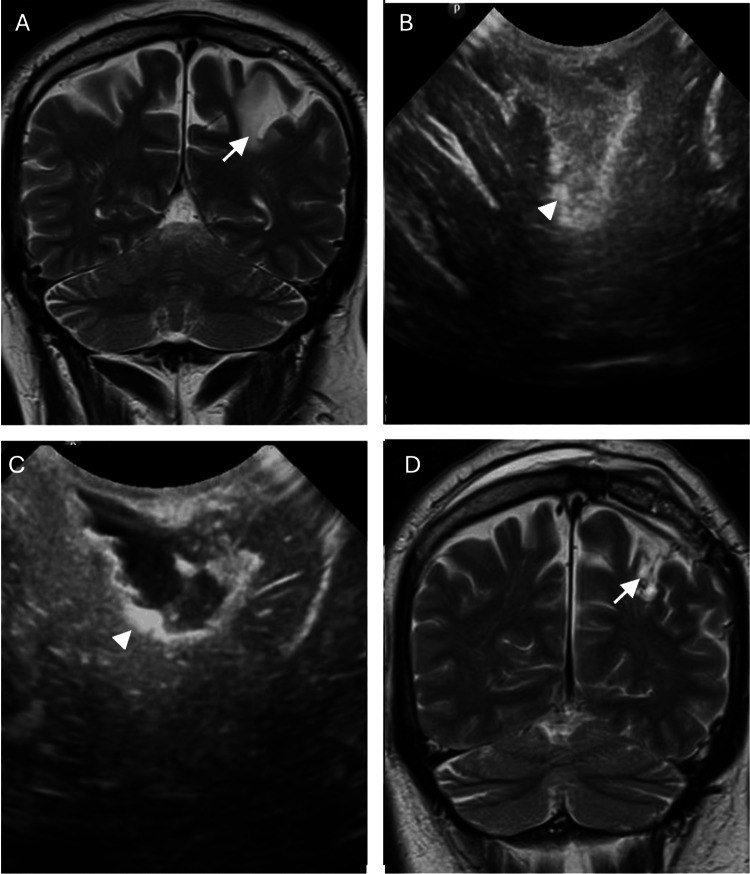
Left frontal low-grade glioma (A) Preoperative T2 MRI shows hyperintense intra-axial tumor (white arrow). (B) Intraoperative ultrasound shows hyperechoic intra-axial tumor with well-defined edges (white arrowhead). (C) Postoperative intraoperative ultrasound image shows total resection of tumor (white arrowhead). (D) Postoperative T2 MRI shows total resection of intra-axial tumor (white arrow).

**Figure 2:**
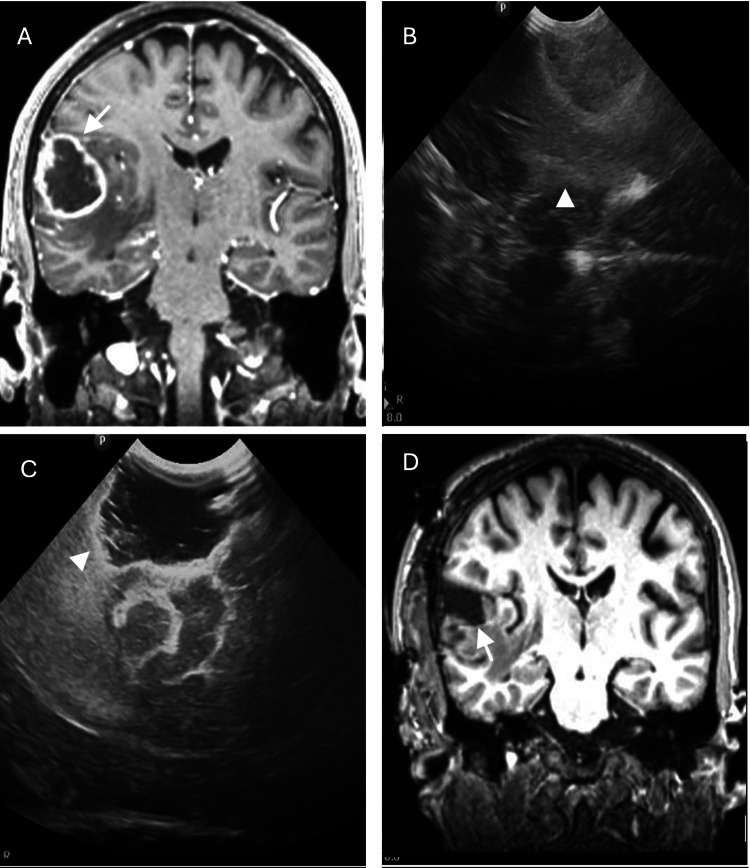
Right temporal high-grade glioma (A) Pre-operative T1 gadolinium-enhanced MRI shows intra-axial tumor with contrast enhancement in the periphery and perilesional edema (white arrow). (B) Intraoperative ultrasound shows intra-axial tumor with central hypoechoic and hyperechoic portions in the periphery, the edges are well defined (white arrowhead). (C) Post-operative intraoperative ultrasound image shows total resection of tumor (white arrowhead). (D) Post-operative T1 gadolinium-enhanced MRI shows total resection of tumor (white arrow).

The limitations of iMRI made the need to have a reliable intraoperative alternative tool. The ultrasound, which has been used since 1982 in neurosurgery, is a reliable tool (Figures 1A-1D, 2A-2D), with the facility of being able to be used intraoperatively. In the last decade, IOUS has been marked by steady technical advancements, increased versatility, wider adoption of technology, improved accuracy, safety, quick and real-time localization of lesions, definition boundaries of the lesion, achieves higher degrees of resection, and reduction of the risk of damage to normal brain structures. It is an accessible low-cost intraoperative tool that does not expose the patient or the surgical team to radiation [7,8,16-20].

The present study was made in TecSalud, the health system at Tecnologico de Monterrey, Mexico. In this case series, we examined the application of IOUS to optimize surgical outcomes by having real-time intraoperative guidance throughout the tumor resection. 

The limitations of iMRI made the need to have a reliable intraoperative tool alternative to iMRI. IOUS, which has been used since 1982 in neurosurgery, has proven to be very useful. In two patients the IOUS evidenced tumor infiltrating eloquent areas, a right frontal glioblastoma with infiltration to the supplementary motor area, and a right temporal glioblastoma with infiltration to the posterior arm of the internal capsule. In both cases, subcortical stimulation was performed that evoked motor response, so the surgeon decided to stop the resection due to the risk of neurological damage, consistent with that reported by Orrigner et al. and Chacko et al. [21,22].

In tumor resection, the cavity is filled with saline irrigation solution and a hyperechogenic artifact at the brain/saline interface is observed. It is very important to differentiate this interface from residual tumor and edema. The attenuation coefficient of saline, which is often used to fill the resection cavity, is lower than that of neural tissue, producing a brightness artifact at the brain/saline interface, and potentially obscuring the identification of residual tumors. While brain tissue itself is relatively homogeneous, the presence of saline, which conducts sound at a slower speed than brain tissue, can produce an error of approximately 1.6 mm at a depth of 10 cm from the transducer. Even the temperature of the saline can change the speed of sound and thereby influence image formation [23]. Coagulated blood or hemostatic materials located along the walls of the resection cavity can also produce a brightness artifact [24]. At the end of the resection, IOUS was useful in searching for residual tumors or hematomas at the surgical site.

In a patient with a mature teratoma in the thoracic spine with two independent lesions, the first extradural lesion was completely resected, and the dura was intact, so it was thought that the tumor was completely resected. The IOUS was performed, and it evidenced that there was a second intradural spinal lesion. The dura was opening, and resection of the second lesion was completed. The IOUS showed the second intradural lesion, allowing complete resection. In a study carried out by Chacko et al., it was reported the extent of resection shown by IOUS at the end of resection correlates with postoperative MRI [22]. Notably, the ability of intraoperative US to accurately delineate the tumor border was equivalent to that of T2-weighted MRI and better than that of T1-weighted MRI [25].

The behavior of brain tissue with IOUS is as follows: cerebral cortex and the gray nuclei appear with a hyperechoic signal, subcortical white matter with a hypoechoic signal, the cerebrospinal fluid with a hypoechogenic signal, the edema presents an intermediate echogenicity between the cerebral cortex and subcortical white matter and a more hyperechogenic signal appears than the subcortical tissue, the choroid plexus appears hyperechogenic and the blood vessels appear hyperechogenic. As reported by Coburger et al. and Unsgaard et al., the echogenicity of a tumor depends on the cell density, the higher the cell density, the greater the echogenicity [12,25]. 

In our case series, we compared the degree of resection by IOUS and postoperative MRI for all extent of resection categories (total, subtotal, and partial), and a concordance of 97.4% was obtained. With P values <0.001 for all observations indicating that the degree of agreement was not random. This makes valid the use of IOUS to localize, plan the surgical approach, guide the resection, verify the degree of resection, and stop the resection of lesions to avoid brain damage and ensure patient safety. Measures of the extent of resection with the use of IOUS correlated highly significantly with postoperative MRI results, indicating that IOUS is an effective adjunctive tool in tumor resection. Using IOS did not confer any identifiable complications and contributed minimal additional time to surgery. A study by Sweeney et al. showed that the use of IOUS may allow for a reliable imaging modality to achieve a more successful GTR of brain tumors in both adult and pediatric neurosurgical patients [26]. The study analyzed by Pino et al. shows a great correlation between postoperative MRI and IOUS, especially for gliomas and metastases [11]. In gliomas, it has been shown that the degree of resection is a significant predictor of overall survival and IOUS allows to increase in the degree of resection, more radical resections thereby increasing overall survival [7,8,11,27-29]. The IOUS is a tool that combined with neuronavigation facilitates tumor removal, enhancing more radical resection, and requires a learning curve, which is fast and does not represent a risk for the patient [13]. As reported by Smith et al., despite the high correlation of IOUS with the postoperative MRI in our case series, IOUS should not supplant postoperative MRI [30]. Postoperative MRI is necessary in all tumor cases. IOUS is an intraoperative tool that provides real-time information on how much tumor remains to decide whether to continue or stop surgery and continuously adjust surgery plans based on the benefit/risk ratio. The greatest utility of IOUS is during the resection of brain lesions, as it allows it to guide the resection of the lesion, achieve a higher degree of resection, and reduce the risks of neurological damage.

Limitations

We note several limitations of our study. We had a small sample, and it is a single-center study, all the IOUS was made for the same neurosurgeon who made a steep learning curve. Further research will be necessary to characterize and correct artifacts that currently limit the use of IOUS in neurosurgical applications and to clarify the value of newer US modalities for achieving maximal safe resections. Despite these limitations, our work provides a successful case study and framework for similar IOUS use in the future.

## Conclusions

In neoplastic and non-neoplastic spinal and brain lesions, IOUS proved to be a useful tool to achieve a higher degree of resection with a lower risk of causing neurological damage. The concordance of the degree of resection by ultrasound and degree of resection by postoperative magnetic resonance is high. IOUS allows the lesions to be characterized and classified by the Mair method. The higher degree of concordance between IOUS and iMRI would suggest that IOUS is a reliable tool for assessing the extent of tumor resection during surgery and provides real-time information to make decisions to adjust surgery plans based on the benefit /risk.
